# Retroperitoneal Ectopic Pregnancy: Diagnosis and Therapeutic Challenges

**DOI:** 10.1155/2017/9871865

**Published:** 2017-10-22

**Authors:** Salma Ouassour, Abdelhai Adib Filali, Mohamed Raiss, Rachid Bezad, Zakia Tazi, Mohamed Hassan Alami, Jihane Bennani, Rachida Dafiri

**Affiliations:** ^1^Department of Obstetrics and Gynecology, National Center for Reproductive Health Ibn Sina, University Mohammed V Rabat, 1 Rue Soekarno, Rabat, Morocco; ^2^Department of Visceral Surgery C, Ibn Sina Public Hospital, University Mohammed V Rabat, Rabat, Morocco; ^3^Department of Radiology, Ibn Sina Public Hospital, University Mohammed V Rabat, Rabat, Morocco

## Abstract

**Background:**

Retroperitoneal ectopic pregnancy is extremely rare. This unusual location represents a great challenge for clinicians due to the difficulties of diagnosis and high risk of life-threatening complications.

**Case Report:**

We report the case of a spontaneous early pregnancy of undetermined location in a patient with a history of previous laparoscopic surgery. Diagnosis steps using clinical examination, ultrasound, and magnetic resonance imaging led to the localization of the pregnancy, in the left side of the para-aortic region, in the retroperitoneal space.

**Conclusion:**

Retroperitoneal ectopic pregnancy is an uncommon entity with rather complex pathogenesis. Clinicians should carefully interpret clinical signs, biological findings, and imaging features and be aware of unusual locations such as the retroperitoneum for ectopic pregnancies. Early diagnosis and appropriate management strategy are conditio sine qua non for successful treatment outcomes.

## 1. Background

Ectopic pregnancy is defined by the implantation of the fertilized ovum outside the endometrial cavity. It is a major cause of maternal mortality during the first trimester of pregnancy and counts for 6% of all maternal deaths, with a maternal mortality rate reaching 0.5 per 1000 cases [1, 2]. Most ectopic pregnancies are located in the fallopian tubes (95%), while the ovaries and abdominal cavity are less frequently involved (1.3%) [3]. Abdominal pregnancies have been classified as either primary or secondary after tubal fertilization and occur in up to 2% of ectopic pregnancies [1].

The retroperitoneal space is an exceptional location for ectopic pregnancies [4]. To date, less than 25 well-documented cases of primary retroperitoneal pregnancy implantations have been reported worldwide, with a history of both spontaneous conception and conception through assisted reproductive technologies (intrauterine insemination (IUI) and in vitro fertilization-embryo transfer (IVF-ET)) [5, 6].

Diagnosis is based on clinical, biological (β-hCG assay values), and ultrasound features. Magnetic resonance imaging (MRI) may prove useful in thoroughly locating the pregnancy and guiding its surgical management but is costly and may not be always available in resource-limited settings [5].

We report the case of an early first trimester retroperitoneal pregnancy occurring after spontaneous conception.

## 2. Case Presentation

A 35-year-old woman, gravida 4, para 2, was referred to our department with a history of 7-week amenorrhea and a positive pregnancy blood β-hCG assay performed for routine antenatal care. Her obstetric history included a laparoscopic salpingectomy for left tubal pregnancy two years ago.

The patient was completely asymptomatic, with stable hemodynamic parameters. Clinical examination was normal: no vaginal bleeding was found, and no lower abdominal or adnexal pain was perceived at combined vaginal examination along with abdominal palpation. Blood β-hCG assay levels were 29386 mUI/ml and then rose to 45057 mUI/ml three days later, while hemoglobin levels were within the normal limits.

Transvaginal sonography at 7 weeks showed a mass of the right ovary measuring 18 mm. The uterine cavity was free of gestational sac, and no adnexal mass on the left side or fluid in the pouch of Douglas was found.

The clinical, biological, and imaging features thus far led to the suspicion of an ovarian pregnancy, for which a Pfannenstiel laparotomy was performed (lack of laparoscopic equipment). While exploring the pelvic cavity, we found a slightly enlarged and soft uterus with a missing left tube, removed from the isthmus. Both the right adnexa and left ovary were normal with no evidence of lesion or of pelvic adhesion. No fluid was found in the peritoneum. No evidence of ectopic intraperitoneal pregnancy could be found, for which we decided to resume the surgical exploration.

We concluded that the ovary mass was rather a corpus luteum, and there was no pelvic pregnancy.

In the following day, a repeated pelvic ultrasound confirmed the presence of the corpus luteum. Moreover, blood levels of β-hCG continued to rise, reaching 60000 mUI/ml in the third day after surgery.

**Figure 1:**
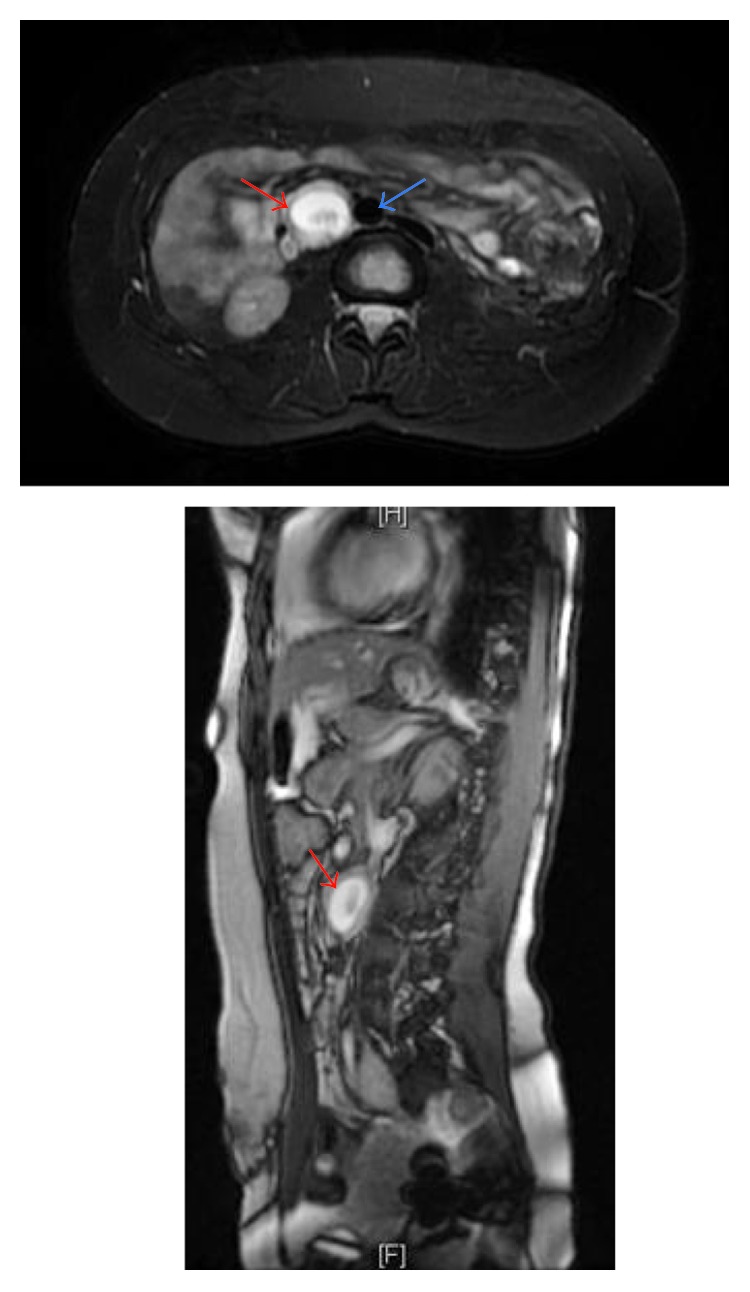
MRI scan in T2 mode showing the gestational sac (red arrow) in the left para-aortic (blue arrow) region.

We were facing the complex situation of an evolutive pregnancy given the β-hCG dynamics, in absence of pelvic pregnancy. We then performed an abdominal ultrasound that revealed a large mass in the left para-aortic region. The mass consisted of a gestational sac with an embryo with positive cardiac activity. We completed with an MRI that showed the gestational sac with the embryo and detailed its tight link with the great vessels alongside (Figure 1()).

**Figure 2:**
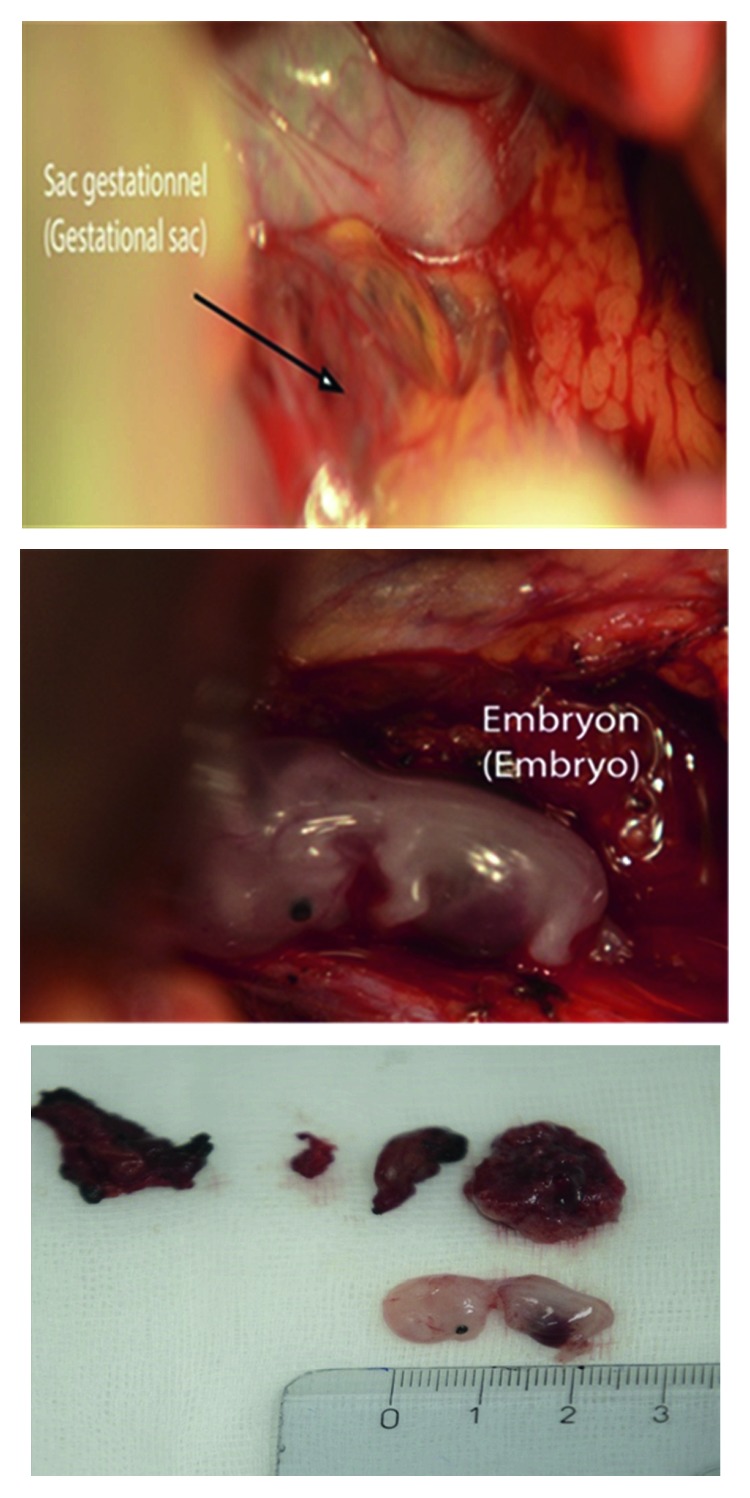
Gestational sac and embryo.

The patient was then scheduled for an exploratory laparotomy by a multidisciplinary team composed of a gynecologist and an abdominal surgeon. The posterior peritoneum was intact. There was no fluid in the peritoneum and retroperitoneum. The retroperitoneal space was carefully dissected, revealing the oval mass of 6 cm attached to the left side of abdominal aorta (Figure 2()) that corresponded to a gestational sac with the embryo. The sac was accidentally cracked but could be removed, along with the embryo of 20 mm and its trophoblastic tissue (Figure 2()). Minimal blood loss was reported, and hemostasis was ensured by bipolar diathermy.

Histopathology examination revealed the presence of decidual tissue and a normal embryo with gestational sac and chorionic villi.

We decided not to administer systemic methotrexate postoperatively, as removal of the trophoblastic tissue appeared complete. Indeed, levels of blood β-hCG declined steeply postoperatively from 731 mIU/ml at day 1 to 55 mIU/ml at day 7 post surgery, indicating complete removal of trophoblastic tissue.

Postoperative period was uneventful, and the patient left on the 7th day. Blood β-hCG levels were measured weekly and reached the undetectable threshold at the twenty-fifth postoperative day.

## 3. Discussion

Most ectopic pregnancies are located in the ampullary segment of the fallopian tube. However, they may also occur in other less common locations, including the interstitial portion of the fallopian tube, the uterine cervical canal, between the leaves of the broad ligament, in the ovary, within a cesarean section scar, or in the abdomen. These unusual locations are difficult to diagnose and are associated with significant morbidity and mortality [1, 2, 4].

There appears to be an increased rate of ectopic pregnancies after assisted reproductive technology (ART) when compared to rates in spontaneous pregnancies. As the number of in vitro fertilization (IVF) procedures performed continues to rise, the incidence of ectopic and abdominal ectopic pregnancies will likely rise along [7].

Abdominal pregnancy is the most rare form of ectopic pregnancy, and its mortality rate is eight times higher than nonabdominal cases [8]. Reported common sites of primary abdominal pregnancy are the pouch of Douglas, the posterior uterine wall, the uterine fundus, the infundibulopelvic ligaments, the anterior abdominal wall, the omentum, the liver, the spleen, the lesser sac, and the diaphragm. In exceptional cases, the embryo implants in the retroperitoneal space [4, 9].

Abdominal pregnancies are classified as either primary or secondary. Most abdominal pregnancies were likely tubal or ovarian pregnancies that ruptured into the peritoneal cavity and implanted elsewhere for a second time.

Only a very small fraction of the reported cases meet the three criteria for primary abdominal pregnancy established by Studdiford [10]: normal tubes and ovaries, absence of uteroperitoneal fistula, and pregnancy related exclusively to the peritoneal surface and diagnosed early enough to exclude the possibility of secondary implantation after primary nidation elsewhere.

The pathogenesis of primary retroperitoneal pregnancy is complex and still unelucidated. However, several explanatory theories have been suggested. Dissemination of cells or tissue fragments through vascular channels, as in the case of trophoblastic diseases, typically terminates in pulmonary tissue, whereas dissemination of endometrial cancers through lymphatic channels leads to metastases in the periaortic and portal hepatic nodes [11]. Hall et al. [12] suggested that the fertilized ovum reaches the retroperitoneal space via the lymphatic system because they found lymphatic tissue together with the ectopic mass. Another explanation advanced was that the embryo implants on the posterior peritoneal surface in the first instance and reaches a retroperitoneal position by subsequent trophoblastic invasion through the peritoneum [13].

A systematic review demonstrated several trends among reported cases of abdominal ectopic pregnancy [7]:

- The history of tubal ectopic pregnancy was particularly common, being reported in 37% of all abdominal ectopic cases.
- The history of prior tubal surgery was also particularly common (50%) among cases.

Our patient conceived spontaneously but had a history of a left tubal ectopic pregnancy managed by laparoscopic salpingectomy.

The preoperative diagnosis of retroperitoneal pregnancy represents the major challenge for surgeons. The main tool in the diagnostic of an early abdominal (and retroperitoneal) pregnancy is transvaginal ultrasonography in conjunction with a quantitative serum human chorionic gonadotropin (β-hCG) test. If early retroperitoneal pregnancy is located outside the pelvis, transvaginal ultrasound examination is helpless, and the diagnosis can easily be overlooked. Other diagnostic tools, such as magnetic resonance imaging (MRI) and other imaging techniques, must be applied [14].

Our case demonstrated the diagnosis challenge of abdominal ectopic pregnancy, as the patient's β-hCG values followed a normal rise and the patient remained asymptomatic up to the point of an evaluation laparotomy that failed to locate the pregnancy.

These facts led us to suggest the possibility of an ectopic abdominal pregnancy at first. So, we did abdominal ultrasonography that suspected the retroperitoneum pregnancy, and furthermore MRI examination clarified the gestational sac with the embryo and showed its tights with the great vessels.

There are no well-defined guidelines for the clinical management of retroperitoneal ectopic pregnancies [15].

Most reported cases of retroperitoneal pregnancies are located close to large blood vessels. The traditional management involves a laparotomy with removal of the embryo with or without placental tissue. Laparoscopic management has not been used very much because control of hemorrhagia due to trophoblastic invasion of retroperitoneal vascular structures can be difficult [16].

The laparoscopic approach is feasible and should be the treatment of choice in hemodynamically stable patients without signs of rupture. Before attempting laparoscopic management of such cases, exclusion of large retroperitoneal vascular infiltration with the assistance of MRI may be necessary, especially in more advanced gestations [5]. Nevertheless, there have been cases reported with laparoscopic management, which was associated with a shorter operative time and reduced blood loss [17].

Any gynecologist attempting such a procedure should be well trained, have a thorough knowledge of the retroperitoneal anatomy, and be ready to convert to laparotomy in case of complications or heavy bleeding. Close cooperation with an abdominal surgeon and/or an interventional radiologist is considerably encouraged to safely carry out these procedures [5].

Adjuvant treatment with methotrexate (systemic treatment or selective arterial embolization) has been suggested to control the risk of heavy bleeding from the placental bed and to avoid the possibility of persistent trophoblastic tissue [1, 17].

Although surgery remains the mainstay of treatment for abdominal ectopic pregnancies, there are also case reports of early abdominal pregnancies being treated successfully with systemic methotrexate, leading to its resorption with no need for further surgery [18].

Our patient underwent an exploratory laparotomy with successful removal of the trophoblastic tissue and minimal blood loss. There was no need to use adjuvant treatment with methotrexate as complete removal of the product was ensured.

## 4. Conclusion

Retroperitoneal ectopic pregnancy represents a great challenge to surgeons due to the rarity and varying clinical presentations, from asymptomatic patients to patients with unstable hemodynamics, in cases of advanced ruptured ectopic gestation presenting with life-threatening retroperitoneal hemorrhage. Laparotomy, laparoscopy, and medical treatment with methotrexate have all been used in the treatment of retroperitoneal pregnancies of various locations. However, each case should be discussed and managed individually.

This case report highlights the diagnosis challenges behind this rare form of ectopic pregnancy and the need to keep it in mind in atypical ectopic pregnancies, especially with normal pelvic ultrasound.

## References

[1] Barnhart K. T. (2009). Ectopic pregnancy. *New England Journal of Medicine*.

[2] Onan M. A., Turp A. B., Saltik A., Akyurek N., Taskiran C., Himmetoglu O. (2005). Primary omental pregnancy: case report. *Human Reproduction*.

[3] Sunday-Adeoye I., Twomey D., Egwuatu E. V., Okonta P. I. (2011). A 30-year review of advanced abdominal pregnancy at the Mater Misericordiae Hospital, Afikpo, southeastern Nigeria (1976–2006). *Archives of Gynecology and Obstetrics*.

[4] Lee J. W., Sohn K. M., Jung H. S. (2005). Retroperitoneal ectopic pregnancy. *American Journal of Roentgenology*.

[5] Protopapas A., Akrivos N., Athanasiou S., Chatzipapas I., Domal A., Loutradis D. (2014). Ultrasound-assisted intraoperative localization and laparoscopic management of a previously missed unruptured retroperitoneal ectopic pregnancy. *Gynecological Surgery*.

[6] Reid F., Steel M. (2003). An exceptionally rare ectopic pregnancy. *British Journal of Obstetrics and Gynaecology*.

[7] Yoder N., Tal R., Martin J. R. (2016). Abdominal ectopic pregnancy after in vitro fertilization and single embryo transfer: a case report and systematic review. *Reproductive Biology and Endocrinology*.

[8] Atrash H. K., Friede A., Hogue C. J. (1987). Abdominal pregnancy in the United States: frequency and maternal mortality. *Obstetrics and Gynecology*.

[9] Chang Y. L., Ko P. C., Yen C. F. (2008). Retroperitoneal abdominal pregnancy at left parcolic sulcus. *Journal of Minimally Invasive Gynecology*.

[10] Studdiford W. E. (1942). Primary peritoneal pregnancy. *American Journal of Obstetrics and Gynecology*.

[11] Yabushita H., Shimazu M., Yamada H. (2001). Occult lymph node metastases detected by cytokeratin immunohistochemistry predict recurrence in node-negative endometrial cancer. *Gynecologic Oncology*.

[12] Hall J. S., Harris M., Levy R. C., Walrond E. R. (1973). Retroperitoneal ectopic pregnancy. *An International Journal of Obstetrics and Gynaecology*.

[13] Ferland R. J., Chadwick D. A., O’Brien J. A., Granai C. O. (1991). An ectopic pregnancy in the upper retroperitoneum following in vitro fertilization and embryo transfer. *Obstetrics and Gynecology*.

[14] Kutlešić R. M., Lukic B., Kutlesic M. (2017). Unruptured retroperitoneal pregnancy implanted in the left broad ligament: a case report. *Vojnosanitetski Pregled*.

[15] Jiang W., Lv S., Sun L., Singer G., Xu C., Lu X. (2014). Diagnosis and treatment of retroperitoneal ectopic pregnancy: review of the literature. *Gynecologic and Obstetric Investigation*.

[16] Martinez-Varea A., Hidalgo-Mora J. J., Paya V., Morcillo I., Martin E., Pellicer A. (2011). Retroperitoneal ectopic pregnancy after intrauterine insemination. *Fertility and Sterility*.

[17] Chetty M., Elson J. (2009). Treating non-tubal ectopic pregnancy. *Best Practice & Research Clinical Obstetrics & Gynaecology*.

[18] Okorie C. O. (2010). Retroperitoneal ectopic pregnancy: is there any place for non-surgical treatment with methotrexate?. *Journal of Obstetrics and Gynaecology Research*.

